# Microstructure, Phase Formation and Heat-Treating of Novel Cast Al-Mg-Zn-Cu-Si Lightweight Complex Concentrated Aluminum Based Alloy

**DOI:** 10.3390/ma15093169

**Published:** 2022-04-27

**Authors:** Spyridon Chaskis, Eva Stachouli, Evangelos Gavalas, Marianthi Bouzouni, Spyros Papaefthymiou

**Affiliations:** 1 Laboratory of Physical Metallurgy, Division of Metallurgy and Materials, School of Mining and Metallurgical Engineering, National Technical University of Athens, 9, Heroon Polytechniou Street, 15780 Athens, Greece; mm16045@central.ntua.gr (E.S.); spapaef@metal.ntua.gr (S.P.); 2 Technology, Marketing and R&D Department of HALCOR, Copper Tubes and Alloys Extrusion Division, ELVALHALCOR S.A., 62nd km Athens-Lamia National Road, 32011 Oinofyta, Greece; 3 Department of Physical Metallurgy and Forming, Hellenic Research Centre for Metals (ELKEME S.A.), 61st km Athens-Lamia National Road, 32011 Oinofyta, Greece; egavalas@elkeme.vionet.gr (E.G.); mbouzouni@elkeme.vionet.gr (M.B.)

**Keywords:** complex concentrated alloys, aluminum-based alloys, high-entropy alloys, CALPHAD, mechanical properties, heat-treatment

## Abstract

In the current work, a novel complex concentrated aluminum alloy is designed and studied. In order to investigate the unknown region of the multicomponent phase diagrams, thermo-physical parameters and the CALPHAD method were used to understand the phase formation of the Al_58_Mg_18_Zn_12_Cu_5_Si_7_ at.% (Al_47_._4_Mg_13_._3_Zn_23_._8_Cu_9_._6_Si_6_wt.%) alloy with a low-density of 2.63 g/cm^3^. The CALPHAD methodology showed good agreement with both the investigated microstructure and the thermodynamic parameters. The designed alloy was manufactured using an induction furnace and pour mold casting process. This study avoids the use of expensive, dangerous or scarce alloying elements and focuses instead on the utilization of widely available relatively cheaper elements. The microstructural evolution as a function of the heat-treatment was studied by means of different microstructural characterization techniques. The hardness, compressive strength and electrical conductivity of the as-cast and heat-treated alloy at room temperature were studied and correlated with the previously characterized microstructure. The alloy is characterized by a multiphase microstructure with major α-Al matrix reinforced with various secondary phases. In terms of mechanical properties, the developed alloy exhibited a high hardness value of 249 Vickers and compressive strength of 588 MPa. The present work provides a valuable insight for researchers, who aim to design and produce industry-like Aluminum based complex concentrated alloys (CCAs).

## 1. Introduction

Traditional alloy design has been based on using one or two main alloying elements with the addition of secondary microalloying elements. Τhe scientific knowledge concerning systems designed to abide by such principles is fully developed., but very little knowledge is available regarding the behavior of systems that are based on the effects that govern alloys with multiple principal alloying elements [1]. Therefore, moving past the traditional restrictive alloy design practices is fast gaining higher importance in the light of increasing environmental, economic and sustainability challenges.

The concept of high-entropy alloys (HEAs) has emerged in the last decades and is based on the theory that an increase of alloying elements would increase the configurational entropy of such systems and promote solid-solution (SS) structures [2].

The limitation of single-phase SS microstructures is surpassed since the term of complex concentrated alloys (CCAs) [3,4] was introduced. Multiphase HEAs, known as CCAs [4,5], are mainly composed of two or more phases, share similar core effects with HEAs and are a subject of research [6]. In this paper, the authors focused on the most recent approach of using the term CCAs which enables a greater degree of freedom in the design and development of novel composition with a more complex microstructure, which could provide broader potential comparing to HEAs.

Such alloys are manufactured by vacuum arc melting and induction melting techniques in Ar or N_2_ protective atmosphere and casting in a water-cooled copper mold [7]. In order to upscale to an industrial scale, the understanding and the tuning of the parameters (melting point, boiling point of alloying elements, castability, liquidity) has become the new challenge, due to the high complexity of the process [8,9,10].

The majority of industry avoids utilization of bulk high-purity raw materials at an industrial plant, since it increases production expenses [11,12]. Implementing industrial-grade purity raw materials to capitalize on circular economy and create added value from lower purity materials is going to help making such alloys viable [13,14,15]. Since the cost of the product is the major driver for manufacturing commercial alloys, the focus point of the current project is turned into designing alloys with economic viable raw materials.

Generally, alloys with density lower than 3 g/cm^3^ are considered lightweight materials [9]. Such alloys are Aluminum-based, which are also popular for their low cost, recyclability and widely availability. Alloy developed in this work will be compared with casting Al-alloys of the series 3xx, 7xx [16], since it was designed combining the elements of those groupings, introducing higher entropy value on the system.

There have been several studies of promising Light Weight HEAs (LWHEAs). Youssef et al. [17] studied a lightweight Al_20_Li_20_Mg_10_Sc_20_Ti_30_ at.% alloy produced by mechanical alloying, which exhibits a combination of high hardness of 591 HV and low density of 2.7 g/cm^3^ that is not equaled by any other metallic material. Furthermore, Tseng et al. [18] designed Al_20_Be_20_Fe_10_Si_15_Ti_35_ at.% alloy, which shows a very high hardness (911 HV) and density of 3.9 g/cm^3^. This alloy’s strength was estimated to be 2976 MPa, which is clearly superior to those of traditional structural materials and had excellent oxidation resistance at 700 °C and 900 °C, which far exceeds that of Ti-6Al-4V.

Specifically on the aluminum-based approach, Gobernik et al. [19] designed and studied cast Aluminum based HEAs. Their goal was to create a castable alloy, which would offer higher specific tensile strength than traditional Al-alloys, while being cost effective. Li et al. [20] focused their studies on the Al-Zn-Li-Mg-Cu system and designed a high-Zn-content Al_80_Zn_14_Li_2_Mg_2_Cu_2_ at.% alloy which undergoes a precipitation transformation where Zn and Al–Zn precipitates in the as-cast state transform into Al_3_(Li,Mg), MgZn_2_ and Zn nano-precipitates after rolling. The precipitation transformation phenomenon contributes to high strength and high deformability. In addition, Li and Zhang [21] focused on designing LWHEAs that were based on Al-Li-Mg-Zn-Sn system with densities varying from 4.23 to 3.08 g/cm^3^ and secondly on Al-Mg-Zn-Cu-Si system. Gondhalekar [22] developed aluminium-based CCAs using the high-entropy alloy concept and specifically those alloys are Al_73.6_Mg_18_Ni_1.5_Ti_1.9_Zr_1_Zn_4_ at.%, Al_65.65_Mg_21.39_Ag_10.02_Ni_2.94_ at.% and Al_67.2_Mg_22.09_Ag_10.7_ at.%. On the same spectrum, Yang et al. [23] focused on Al-Li-Mg-Zn-Cu system and Al_80_Li_5_Mg_5_Zn_5_Cu_5_ at.% alloy shown the best properties in that system with a yield strength of 488 MPa and plastic deformation of 18%. Furthermore, Zhang et al. [24] shifted the research on the effects of Zn and Cu additions on the microstructures and properties of medium-entropy alloys (MEAs) based on 5083 aluminum alloy. Li et al. [25] designed lightweight multicomponent alloys with compositions of Al_(86-x)_Mg_10_Zn_2_Cu_2_Si_x_ (x = 0, 0.3, 0.6, 0.9, 1.2 at.%). Sanchez et al. [26] studied the HEAs Al_40_Cu_15_Mn_5_Ni_5_Si_20_Zn_15_ at.%, Al_45_Cu_15_Mn_5_Fe_5_Si_5_Ti_5_Zn_20_ at.%, Al_35_Cu_5_Fe_5_Mn_5_Si_30_V_10_Zr_10_ at.% and Al_50_Ca_5_Cu_5_Ni_10_Si_20_Ti_10_ at.%, which are produced by large scale vacuum die casting and show exceptionally high strength/density ratios. In [5], using industrial-scale die-casting, the authors produced non-equiatomic lightweight MEAs based on the Al_65_Cu_5_Mg_5_Si_15_Zn_5_X_5_ and Al_70_Cu_5_Mg_5_Si_10_Zn_5_X_5_ systems, where X is Fe, Ni, Cr, Mn and Zr. These alloys surpass the commercial Al-alloys in terms of mechanical properties. Furthermore, they manufactured Al_40_Cu_15_Cr_15_Fe_15_Si_15_, Al_65_Cu_5_Cr_5_Si_15_Mn_5_Ti_5_ at.% and Al_60_Cu_10_Fe_10_Cr_5_Mn_5_Ni_5_Mg_5_ at.% HEAs via large scale vacuum-die casting and reported the highest hardness of 916 HV for the Al_40_Cu_15_Cr_15_Fe_15_Si_15_ at.% alloy [4]. In their recent article they produced Al_80_Mg_5_Sn_5_Zn_5_Ni_5_ at.%, Al_80_Mg_5_Sn_5_Zn_5_Mn_5_ at.%, and Al_80_Mg_5_Sn_5_Zn_5_Ti_5_ at.% alloys using a gravity permanent mold casting, while avoiding the alloying with expensive elements and studied the effect of acicular Al_3_Ni, Al_6_Mn and globular Al_3_Ti on the mechanical properties [27]. Finally, Baek et al. [28] studied the effects of ultrasonic melt treatment and solution treatment on low-density multicomponent Al_70_Mg_10_Si_10_Cu_5_Zn_5_ at.% alloy and showed that it was characterized by excellent strength at temperatures below 200 °C, unlike the commercial Al-alloys. To our knowledge, there has been only one study so far, on design of lightweight, low-cost MEAs [29], where AlMgLiCa, Al_2_MgLiCa and AlMgLiCa_0.3_ at.% were manufactured via conventional crucible melting and mold-casting in air atmosphere.

Up to this date, there is still a vast unknown space in low density multicomponent phase diagrams, from the corners of traditional Al-alloys to the center of the equimolar LWHEAs. Therefore, the unexplored region of the phase diagram has been considered. It is widely known that the highest strength and highest application temperatures are met by alloys with a controlled distribution of a secondary phases [30]. Thus, the developed alloy, was designed in order to obtain a major SS phase reinforced with different intermetallics, with a density below 3 g/cm^3^. The main objective of this research is to develop a novel lightweight aluminum based CCA in the system of Al-Mg-Zn-Cu-Si on a more industrially relevant approach.

## 2. Materials and Methods

In order to design HEAs and CCAs, some empirical parameters are taken into consideration. For the prediction of solid-solution phase formation, various empirical approaches based on Hume–Rothery rules and thermodynamic parameters were proposed [12,13]. These criteria consist of parameters such as the enthalpy of mixing in the liquid phase (ΔHmix), atomic size difference (r), Pauling electronegativity difference (Δχ) and the Ω parameter. These standalone parameters are not sufficient conditions to form single-phase solid-solution phases [14]. They are, however, useful as preliminary design indicators to define an alloy system with a percentage of error. In this study, the artificial neural network software pyMPEALab Toolkit (Upadesh Subedi et al.) was utilized for the thermodynamic calculations, in order to calculate the thermophysical parameters of the chosen alloy [31]. The Thermocalc® software (Version 2022a, Thermo-Calc Software AB, Stockholm, Sweden) in conjunction with the TCAL07 thermodynamic database was used for calculation of the phase diagram. This tool shows the equilibrium phases as a function of temperature, in order to provide a rough indication of the type of phases that would form on the selected alloy [32].

Experimental alloy was prepared in an induction furnace in an alumina crucible using industrial purity materials, without any protective atmosphere. Casting flux was used to protect the melt pool surface and prior to casting the molten metal was skimmed. To be more specific, the ingot was manufactured by a typical furnace pour mold casting method similar to the industry practice. Master alloys of Al-Cu and Al-Si containing 35 wt.% of Cu and 12.5 wt.% of Si respectively were prepared in advance. The purpose of the master alloys at eutectic composition, is to reduce the overall melting point. Without which, alloying would be difficult due to the presence of easy to oxidize elements such as Mg or elements that have a boiling point lower that the melting point of other alloying elements (e.g., Zn in comparison to Si). Both these master alloys are also commercially available.

Physical characteristics for the utilized alloying elements are shown at Table 1. During the casting trial, the Al, AlSi12.5, AlCu35 guarantee a bath base at 800 °C. Subsequently, the Mg and Zn were added to the molten pool and soaked for 1 h. Finally, the melt was cooled down to 700 °C and poured manually into a steel mold (pouring temperature of melting +50 °C superheat).

**Figure 1:**
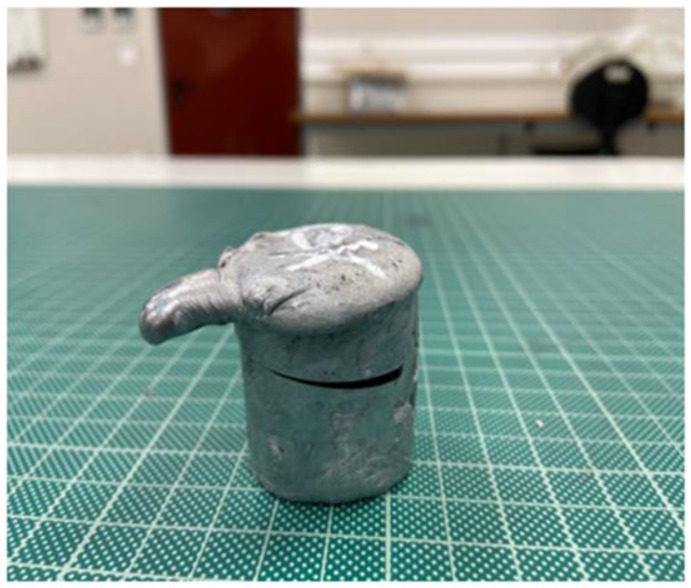
The solidified ingot, which was sectioned in half.

Ingot of 40 mm × 40 mm × 60 mm was obtained as shown below (Figure 1). Following which, the microstructures of as-cast and heat-treated alloy, at room temperature, were studied to evaluate the suitability and behavior of the developed CCA. Two microstructural samples were sectioned from the center of the ingot and prepared according to standard metallographic procedures, by cold mounting in resin, grinding with 80, 220, 500, 1200 grit papers and polishing with cloth of 3 μm (Struers DiaPro Mol B3), 1 μm (Struers DiaPro Nap B1) and 0.04 μm (Struers OP-U NonDry). Specimen 1M originates from the middle of the ingot’s center, while specimen 2M originates from the side of the ingot’s center. Specimens 1MH and 2MH undergo heat-treatment at 400 °C for 24 h with water cooling, while 1MH’ are subjected heat-treatment at 200 °C for 24 h with water cooling and is originated from the same position as 1M. The microstructure, the different regions, and the averaged overall chemical composition of each sample were investigated by an optic microscope model Epiphot 300 (Nikon, Tokyo, Japan) and a JEOL JSM-IT800 scanning electron microscope (SEM), equipped with an energy dispersive X-ray spectrometry (EDS). The X-ray diffraction (XRD) equipment used to characterize the crystal structures of the alloy was a Phillips Xpert diffractometer with a long fine focus copper anode X-ray source and the diffraction diagrams were measured at the diffraction angle 2θ, range from 10° to 110° with a step size of 0.02°/s.

A Vickers hardness Duramin-40 M1 model (Struers, Copenhagen, Denmark) measurer was employed on the polished sample surface using a 0.2 kg load, applied for 10 s. At least 10 random individual measurements were made for each test. At least five specimens were recorded to ensure repeatability. For the measure of electrical conductivity, a SIGMATEST D-2.068 (FOERSTER Instruments Inc., Pittsburgh, PA, USA) device was utilized and at least 5 random individual measurements were made for each specimen. A tensile testing Zwick/Roell Z1200 (ZwickRoell, Ulm, Germany) machine was utilized for the compression testing of samples—prepared as per internal specifications.

## 3. Results-Discussion

### 3.1. Thermo-Physical Parameters for Phase Formation in HEAs/CCAs

The alloy Al_58_Mg_18_Zn_12_Cu_5_Si_7_ at.% has Al as the main alloying element, for the abovementioned reasons, and other elements quantity has to be at least 5 at.% in order to be considered as principal alloying element [33]. Moreover, Cu and Zn were chosen since they are relatively cheap and are elements widely used in the metallurgical sector. From the study of the thermo-physical parameters for phase formation in HEAs, the Al_58_Mg_18_Zn_12_Cu_5_Si_7_ at.% alloy is characterized by the values in Table 2. In this system, a microstructure predominantly formed by Al-base face-centered cubic (FCC) matrix phase with other intermetallics is expected to be stabilized at room temperature. It is important to consider that, it is generally very difficult to keep the ΔHmix values within the limits for solid-solution phase formation [27]. Alloy’s theoretical density was calculated by using the rule of mixture.

### 3.2. CALPHAD Methodology and Equilibrium Phase Diagram

**Figure 2:**
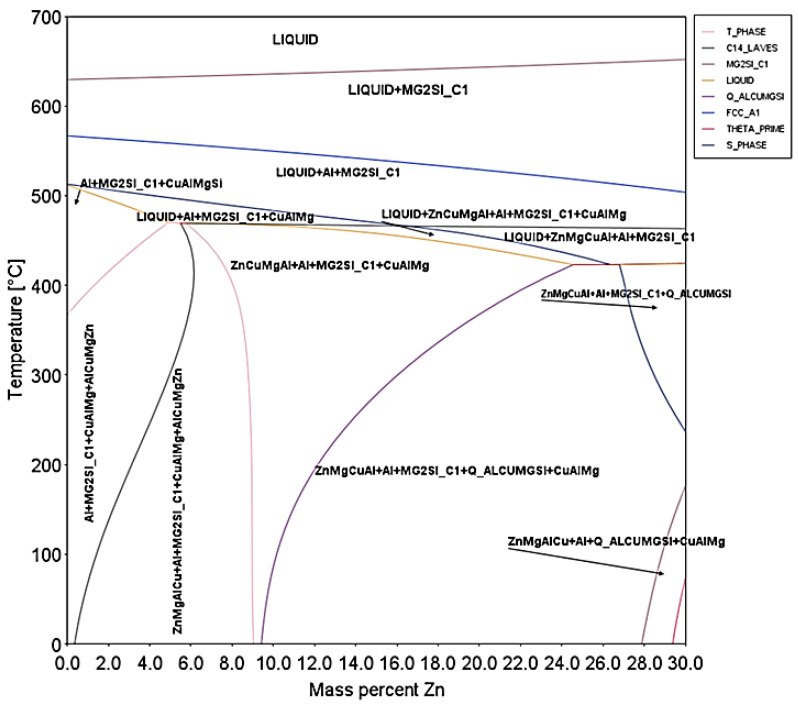
Equilibrium phase diagram of function of Zn content. Arrows represent the areas of thermodynamic stability for various phases.

The CALPHAD methodology is utilized for the construction of equilibrium phase diagram, which is depicted in Figure 2. It provides information for possible phases that can be formed in the alloy and is a useful tool for screening out chemical compositions and aids in alloy development process. Starting with the melt being poured at the mold at melting +50 °C superheat. In reducing the temperature, it is clear that approximately around at 630 °C, the first Mg_2_Si particles start forming. The FCC aluminum-based matrix begin its formation around 530 °C. Around 460 °C, a C14-phase (ZnCuMgAl) nucleates, while 30 °C below S-phase (CuAlMg) will start forming as well. At 420 °C, the melt completely solidifies with the formation of Q-phase (AlCuMgSi).

The designed alloy is composed of a mixture of five different phases at equilibrium state. Hence, it is expected to be composed of at least of FCC (A1), Mg_2_Si (C1), Q-AlCuMgSi, C14 laves-phase (ZnMgCuAl), S-phase (CuAlMg). The Mg_2_Si is a desirable phase to improve the mechanical properties by the formation of intermetallic compounds in various Al-base alloys. It is usually found in plate shapes [34], as blocks-octahedron [35] or truncated octahedron [36], as hopper [36], as dendrite [36], as eutectics [37,38], as dispersoids [39] and even as nano-particles [40,41]. The Q-AlCuMgSi is a stable quaternary phase known as Al_5_Cu_2_Mg_8_Si_6_, Al_3_Cu_2_Mg_9_Si_7_ or Al_4_Cu_2_Mg_8_Si_7_ and it is typical found in some Al-Cu-Mg-Si alloys and it is worthy to mention the fact that Q-phase precipitates for many commercial alloys [34,42,43,44,45,46,47]. The C14 laves-phase is a common stable precipitate in 7000 series aluminum alloys, widely known as the η (MgZn_2_) phase (or M phase) and includes all includes all MgZn_2_ type phases in aluminum alloys, although the volume fraction of it is usually minor and it is found in nano-scale [40]. Moreover, the S-phase is a disordered Cu-containing β′-variant [48], formed at nano-scale [49] and it is usually found in the form of Al_2_CuMg [50]. The major crystallographic information is summarized by Thermocalc® software (Version 2022a, Thermo-calc Software AB, Stockholm, Sweden) and given in Table 3.

### 3.3. Microstructural Characterization and X-ray Diffraction Analysis

**Figure 3:**
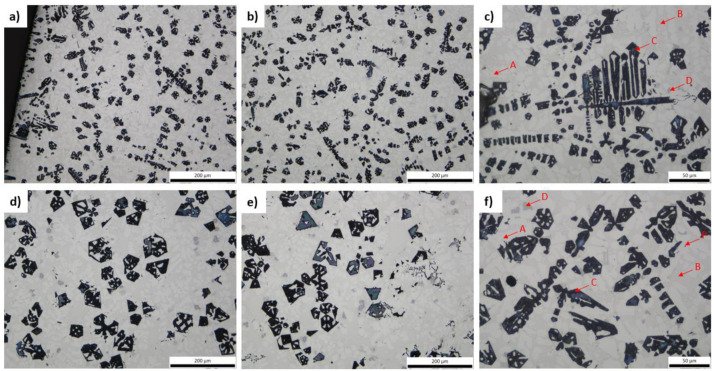
Optical micrographs of 2M (**a**–**c**) and 1M (**d**–**f**).

By studying the as-cast 2M (side of middle section of ingot) and 1M (middle of ingot) the existence of dendritic morphology is obvious (Figure 3). The formation of four phases (A, B, C, D) is evident along with the existence of some pores (P). The dark intermetallic particles, designated as C, appear to be smaller in size at the edge of the ingot than at the center, due to the temperature gradient and cooling rate. The existence of minor shrinkage porosity is evident in the samples, that does not otherwise influence outcome of the present study and is easily addressed in industrial scale. It is worth noting that the angular dark-colored particles appear to form in dendritic morphology in some cases (Figure 3c) and in other cases appear more concentrated or even at the grain boundaries (Figure 3f). It is needed to mention that pores occur near the points of dark angular particles. In total four phases can be observed. Light-white colored dendritic regions, which are probably the aluminum based phase are designated as A, dark intermetallic phases, eutectic regions, designated as B, and very small light-grey particles, designated as D.

**Figure 4:**
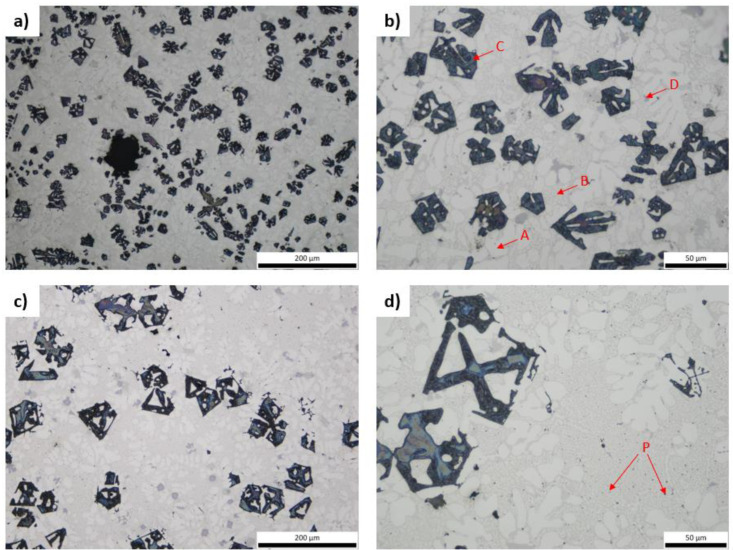
Optical micrographs of 1MH (**a**,**b**) and 2MH (**c**,**d**).

Regarding the heat-treated samples, no significant structure alteration is perceived compared to the corresponding as-cast specimens. It is worth underlining that the angular particles (phase C) do not appear to be affected by the heat-treatment at 400 °C with soaking time of 24 h (Figure 4).

Analysis of the dark-colored compounds took place with Image Pro Plus software (Version 7.0.1.658, Media Cybernetics Inc., Rockville, MD, USA). For specimens 1M and 2M, the average volume fraction of those intermetallics is 19.3% and 17.5%, respectively, with standard deviation of 0.3% and 0.4%. Whereas after heat-treatment at 400 °C for 24 h, the average fraction increases to 24.3% for 1MH and 20.9% for 2MH with standard deviation of 0.4% and 0.2%, respectively. Compared to data from given from Thermocalc®, the produced alloy’s values are not far from the 20.5% predicted by CALPHAD methodology.

**Figure 5:**
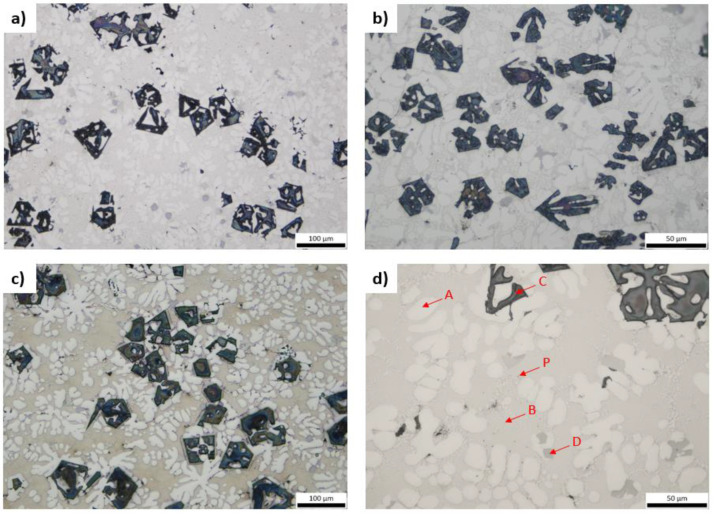
Optical micrographs of 1MH (**a**,**b**) and 1MH’ (**c**,**d**).

Comparing heat-treatment between 400 °C and 200 °C, it is evident that the bright-white phase becomes coarser in shape (Figure 5c). The very small light-grey particles are still present throughout the material. By means of optical microscopy, intermetallic particles seem to be unaffected by heat-treatment (Figure 5c).

The overall composition of the alloys estimated using EDS over large areas is presented in Table 4. It is evident that the minor deviation from the nominal composition is within the ±1 acceptance range of industrial practice. Probably it is attributed to micro-segregation phenomena, since EDS analysis were carried out at the center of the specimen.

**Figure 6:**
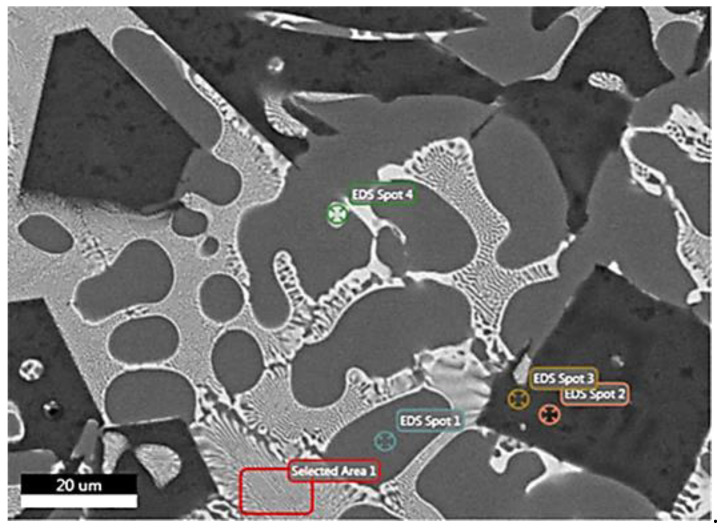
FESEM backscatter electron microstructure with EDS on selected phases for specimen 1M.

Since the microstructure is similar in all the ingot’s geometry, FESEM observations take place for specimen’s 1Μ microstructure in Figure 6. Analysis at Spot 1 verifies the formation of an Al-rich solid-solution matrix, whereas Spot 2 and Spot 3 show the precipitation of primary Mg_2_Si intermetallic compounds (Mg-silicides). It is worth mentioning that very small dark spots on Mg_2_Si compounds contain some oxygen, but it is within the range of error of SEM analysis. Moreover, Spot 4 indicates a phase consisting of ZnMgCuAl. The eutectic structure of alternating dark-bright stripes (Selected Area 1) is composed of ZnMgCuAl and Al-base as shown in Table 5.

Numerous measurements have been conducted in order to ensure the accuracy of chemical composition from a statistical point of view. The Table 6 summarizes the phases and their chemical composition as measured in the alloy by EDS analysis.

**Figure 7:**
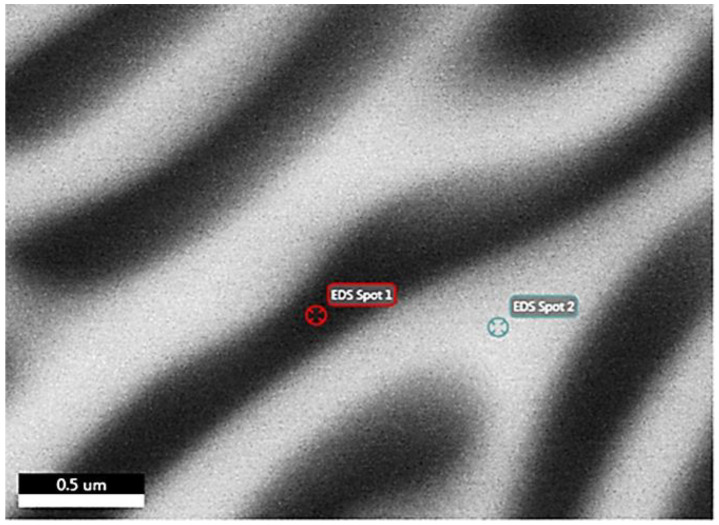
FESEM backscatter electron microstructure of sample 1M in the stripe area.

Observing the striped area at higher magnifications by EDS (Figure 7) confirms an eutectic structure. This structure consists of alterations with higher Al content on black stripes and higher Mg, Cu content on white stripes (Table 7). However, due to the relatively small lamellae of the eutectic phase, which are less than 0.5 μm, the measured phase is probably influenced by the neighboring phase.

**Figure 8:**
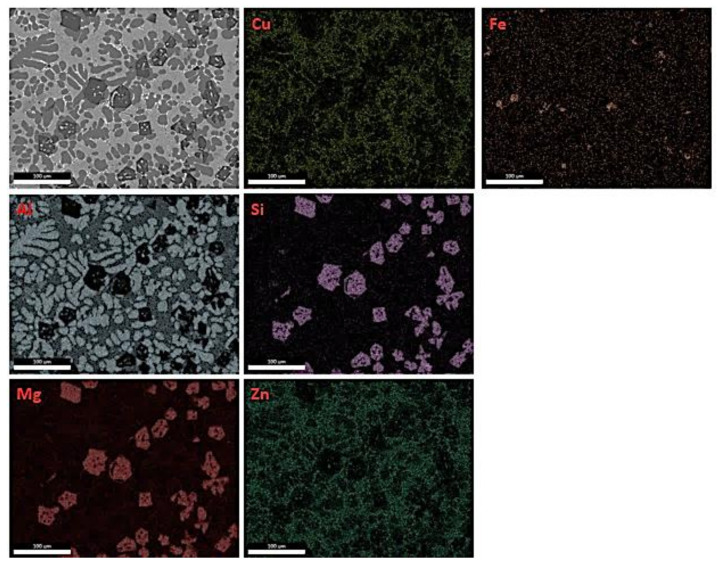
EDS map of 1M specimen.

From the elemental map for the specimen 1M in Figure 8 it is obvious that there is some micro-segregation phenomena in the structure of the cast specimen. The observed large blocks are Mg_2_Si particles. In addition, Fe is present in the elemental map, probably due to the use of industrial purity raw materials.

**Figure 9:**
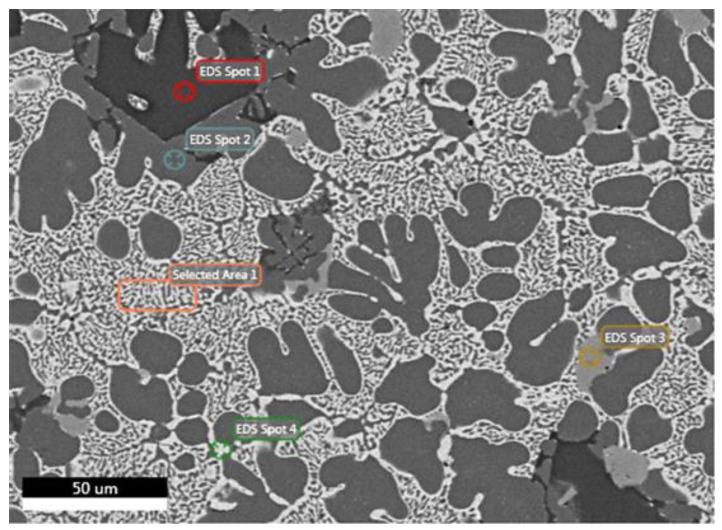
FESEM backscatter electron microstructure with EDS on selected phases for specimen 1MH.

For specimen 1MH, FESEM analysis took place in Figure 9. Analysis at Spot 2 verifies the formation of Al-rich solid-solution matrix, whereas Spot 1 shows the precipitation of primary Mg_2_Si intermetallic compounds. It is worth mentioning that very small dark spots on Mg_2_Si compounds contain very low amount of oxygen, which is within the SEM’s analysis error range. Light-grey areas at Spot 3 are rich in Al, but it seems that Fe and Mn impuritites from scrap tend to concentrate at those areas. Moreover, Spot 4 indicates a phase consisting of ZnMgCuAl. The eutectic structure of alternating dark-bright lamellae (Selected Area 1) is composed of ZnMgCuAl and Al-base as shown in Table 8.

Similar to the as-cast sample, several measurements have been conducted in order to evaluate and guarantee the accuracy of chemical composition from a statistical viewpoint. The Table 9 summarizes the phases and their chemical composition as measured in the alloy by EDS analysis.

**Figure 10:**
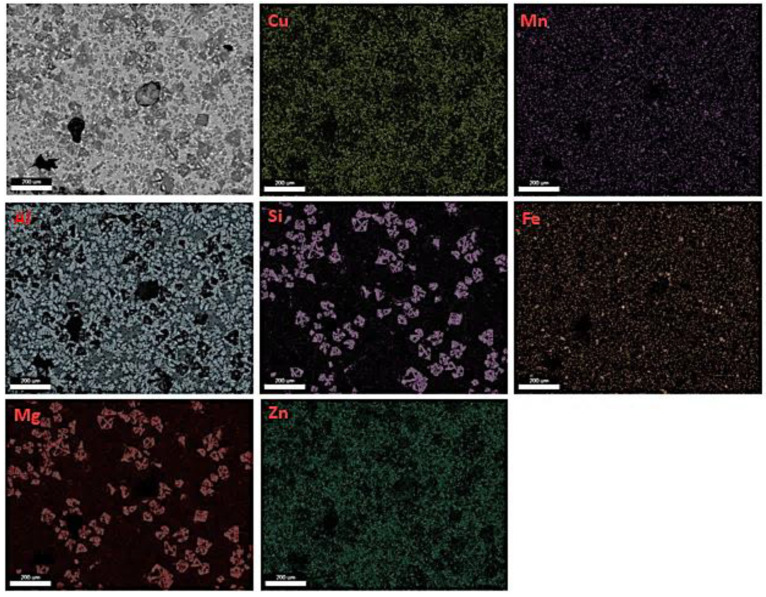
EDS map of 1MH specimen.

From the elemental map for the specimen 1MH in Figure 10 it is obvious that there is some micro-segregation phenomena in the structure of the heat-treated specimen. Some elements like Fe, Mn and O are present, but they are again at very low level, as shown previously.

**Figure 11:**
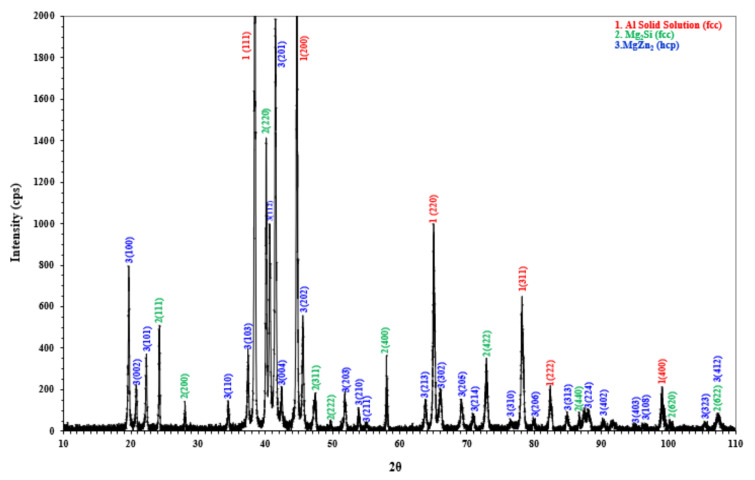
X-ray diffraction pattern of 1MH’ specimen.

Additional, diffraction analysis comes into evaluation of the produced alloys. In Figure 11 indicative XRD analysis of 1MH’ sample is presented. A predominant Al-base phase with an FCC structure can be observed. Second are the coarse Mg_2_Si intermetallic particles of FCC structure and then a MgZn_2_ phase with hexagonal close-packed (HCP) structure. The MgZn_2_ phase is the eutectic phase, which is consisted of ZnMgCuAl. The XRD analysis confirms the results from EDS analysis. The microstructural evolution revealed that CALPHAD overestimates the stability of some phases, since S-phase (CuAlMg) and Q-phase phase were not stabilized or at least could not be quantitively analyzed by means of scanning electron microscopy, whereas Q-phase precipitates form in the nanometer scale for many commercial alloys. However, even in widely used alloys there are some variances from CALPHAD approach.

### 3.4. Mechanical & Physical Properties

**Figure 12:**
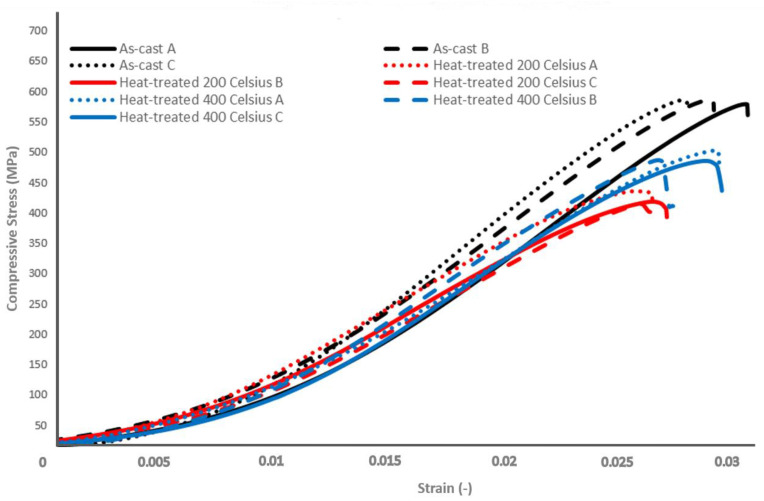
Compressive strength diagram.

In order to investigate and evaluate the mechanical properties of the as-cast and heat-treated alloys, hardness tests were conducted at room temperature as shown in Table 10. The compressive strength of the as-cast and heat-treated alloys was measured at room temperature and the diagram is shown in Figure 12.

The mechanical properties are affected during heat-treatment, since measured hardness and compressive strength decreased. During compression testing all specimens failed in a brittle manner. The as-cast samples shown the highest maximum compressive strength of average 588 MPa. On the contrary, the compressive strength decreases with decreasing heat-treatment temperature. After treatment at 400 °C and 200 °C the strength falls to 495 MPa and to 426 MPa, respectively.

**Figure 13:**
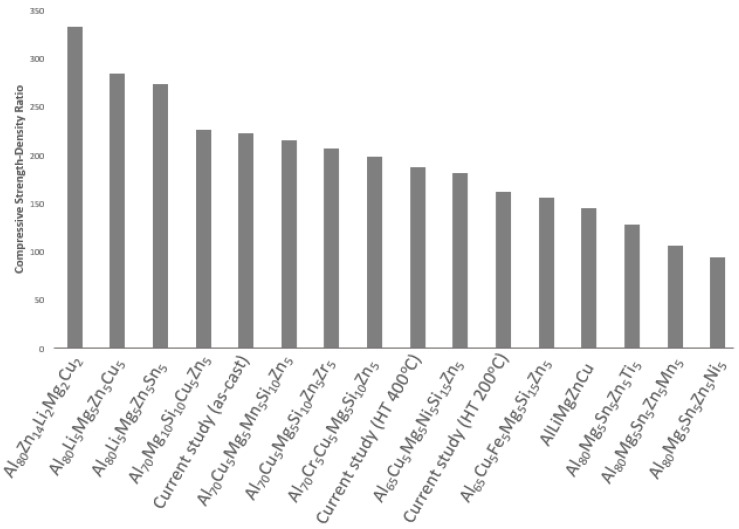
Comparative diagram of compressive strength and density ratio for current study and various alloys in literature [5,20,23,26,27,28].

According to microhardness measurements conducted, after heat-treatment at 400 °C the total hardness decreases by 20%. This is a notable decrease, which can probably be attributed to the fact that the fast cooling of the produced ingot holds elements into solution and after heat-treatment, these elements tend to diffuse and reduce the total hardness. Further indentations have been conducted on Mg_2_Si particles in order to evaluate their hardness with 0.05 kg for 15 s. The mean value of Mg_2_Si microhardness has been calculated to be 385.55 HV0.05. It is more than evident that these particles are harder than the matrix and thus this alloy is characterized by enhanced strength. Additionally, heat-treatment at 400 °C causes a decrease in compressive strength by 15%, whereas heat-treatment at 200 °C decreases compressive strength by 28%. It is evident that the designed alloy shows high hardness–density ratio, specifically when compared to other cast aluminum alloys [53,54], shown in Table 11 as reference. These results are quite promising, since the newly manufactured alloy can be proved as an appealing alternative for applications where commercial cast aluminum alloys are used, since its hardness to density ratio is higher. Furthermore, this new system can be directly compared with some major LWHEAs from the literature. It is evident from diagram in Figure 13 that the designed alloy’s ratio of compressive strength to density is close to the majority of the literature and can offer a lighter alternative to the already proposed low-density aluminum-based HEAs. From an economic point of view, taking into consideration production and cost limitations, Al_58_Mg_18_Zn_12_Cu_5_Si_7_ offers a friendly alternative, since it was manufactured with industrial purity raw materials and with a common furnace. Although there is still a lot of room for exploration in the field of CCAs, the microstructure and the superior strength-to-density ratio of the investigated alloy in combination with the manufacturing method that focuses on the industrial practice can lend it application in the structural, automotive and energy industry. The assessment of particular properties based on the exact needs can confirm the suitability for each specific application.

**Figure 14:**
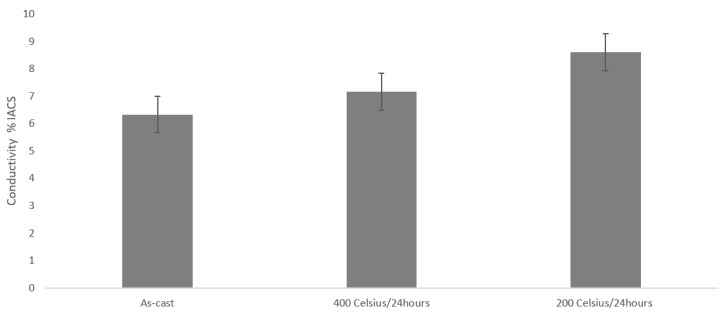
Conductivity measurements of 1M, 1MH and 1MH’ specimens.

Additionally, electrical conductivity measurements were conducted in order to evaluate the different conditions (as-cast and heat-treated) as shown in Figure 14. It is evident after heat-treatment the electrical conductivity increases. In the as-cast specimen it measured 6% IACS, whereas after heat-treatment of 400 °C and 200 °C it was measured as 7% IACS and 9% IACS respectively.

During casting and solidification, alloying elements are trapped in solid-solution since there is limited time for diffusion. The fact that the electrical conductivity increases in both heat-treatments indicates that the elements diffuse/precipitate from solid-solution. Such diffusion phenomena might not be sluggish, like HEAs core effects suggest, since the alterations in mechanical properties after 24 h are major.

The decrease in mechanical properties and hardness after heat-treatment indicates that the most prominent strengthening mechanism is solid-solution hardening. Although at 200 °C there is less energy for kinetics and diffusion, volume fraction of C14-laves gradually increases from solidus all the way to room temperature at the expense of Al-base and Mg_2_Si, reaching the maximum volume fraction at room temperature and thus the minimum degree of solid-solution. The condition of the material after the heat-treatment at 400 °C lies in between the as-cast and heat-treated at 200 °C regarding volume fraction and degree of solid-solution. The minimum hardness and compressive strength after heat-treatment at 200 °C is explained by the fact that the alloy’s principal strengthening mechanism is solid-solution.

## 4. Conclusions

In this work, a new aluminum-based CCA (Al_58_Mg_18_Zn_12_Cu_5_Si_7_ at.%) was successfully developed with a density of 2.63 g/cm^3^ and manufactured by a standard furnace and pour mold casting process. Widely available, low cost raw materials were utilized, enabling the production of new promising industrially feasible alloys. From the equilibrium phase diagram and thermo-physical parameters for phase formation in HEAs, the alloy was expected to be composed of a mixture of solid-solution and intermetallics microstructure.

- There is consistency of CALPHAD and thermo-physical parameters calculations regarding the major Al-base and Mg_2_Si and C14 Laves. CALPHAD approach overestimates the stability of S-phase and Q-phase.
- Scanning electron microscopy and X-ray diffraction analysis confirm the formation of three major phases.
- Mechanical properties achieved are 588 MPa compressive strength for the as-cast specimen, 495 MPa and 426 MPa for heat-treated at 400 °C and 200 °C respectively. Hardness for the as-cast specimen, heat-treated at 400 °C and 200 °C is 249, 200 and 171 Vickers respectively.
- Heat-treatment leads to decrease of hardness and compressive strength and an increase of electrical conductivity.

## Figures and Tables

**Table 1:** Physical information about the utilized alloying elements and master alloys.

Alloying Elements	Melting Temperature (°C)	Boiling Temperature (°C)	Density (g/cm^3^)
Al	660.3	2470	2.7
Mg	650	1091	1.738
Si	1410	2355	2.33
Cu	1085	2562	8.96
Zn	419.5	907	7.133
AlSi_12.5_	577	-	-
AlCu_35_	548.2	-	-

**Table 2:** Thermo-physical parameters and the theoretical density for the developed alloy.

ΔHmix (kJ/mol)	δ (%)	ΔSmix (J/K/mol)	Ω	Δχ	VEC	Τm (K)	ρ_theoretical_ (g/cm^3^)
−4.94	7.64	10.1	2	0.16	4.37	976.06	2.63

**Table 3:** Crystallographic parameters of phases provided by Thermocalc® software and literature [34,44,51,52].

Phases	Chemical Composition (Mole Percent)	Lattice Type	Lattice Parameters(Å)	Symmetry/Space Group
FCC-A1	Al_0.99_	FCC	a = 4.05	$\mathrm{Fm}\bar{3}$m
Mg_2_Si-C1	Mg_0.66_Si_0.33_	FCC	a = 6.351	$\mathrm{Fm}\bar{3}$m
C14-laves	Zn_0.57_Mg_0.33_	HCP	a = 4.9 c = 7.8	$P6\bar{3}$/mmc
S-phase	Cu_0.25_Al_0.5_Mg_0.25_	Orthorhombic	a = 4.01 b = 9.23 c = 7.14	Cmcm
Q-phase	Mg_0.38_Si_0.29_Al_0.24_Cu_0.09_	HCP	a = 10.39 c = 4.02	$P\bar{6}$

**Table 4:** Elemental composition of the manufactured alloy in 1M specimen obtained by EDS.

Alloying Elements	Al	Mg	Zn	Cu	Si
Nominal (at.%)	58	18	12	5	7
Full Area (at.%)	58.2	18.5	12.8	4.5	6.0
Full Area (wt.%)	47.4	13.6	25.3	8.6	5.1

**Table 5:** Elemental composition of the manufactured alloy in sample 1M obtained by EDS.

Analysis/ Elements	Spot 1 (at.%)	Spot 1 (wt.%)	Spot 2 (at.%)	Spot 2 (wt.%)	Spot 3 (at.%)	Spot 3 (wt.%)	Spot 4 (at.%)	Spot 4 (wt.%)	Area 1 (at.%)	Area 1 (wt.%)
O	-	-	0.4	0.2	0.2	0.1	-	-	-	-
Mg	3.8	3.2	67.1	62.8	66	61	19.3	9.8	13.6	8.2
Al	89.9	82.8	-	-	1.3	1.3	24.2	13.6	49.7	33
Si	-	-	31.2	33.7	30.4	32.5	-	-	-	-
Cu	0.9	2	0.3	0.7	0.6	1.4	15	19.9	10.3	16.2
Zn	5.4	12	1	2.6	1.5	3.7	41.5	56.7	26.4	42.6

**Table 6:** Phases composition of the manufactured alloy in sample 1M obtained by EDS.

Elements/Phases	Al-Base (at.%)	Mg_2_Si (at.%)	Eutectic (at.%)
Mg	3.5 ± 1.1	68 ± 2.3	14.4 ± 1.4
Al	90 ± 2	-	48.1 ± 1.8
Si	-	30.8 ± 1.9	-
Cu	1 ± 0.2	0.3 ± 0.3	9.6 ± 1.6
Zn	5.5 ± 1.9	0.9 ± 0.2	27.9 ± 1.7

**Table 7:** Elemental composition of the manufactured alloy in eutectic region of sample 1M obtained by EDS.

Analysis/ Elements	Spot 1 (at.%)	Spot 1 (wt.%)	Spot 2 (at.%)	Spot 2 (wt.%)
Mg	9.4	5.8	16.1	9.3
Al	56.4	38.4	43.5	28.1
Cu	9.2	14.7	11.5	17.5
Zn	24.9	41	28.9	45.1
Si	0.1	0.1	-	-

**Table 8:** Elemental composition of the manufactured alloy in sample 1MH obtained by EDS.

Analysis/ Elements	Spot 1 (at.%)	Spot 1 (wt.%)	Spot 2 (at.%)	Spot 2 (wt.%)	Spot 3 (at.%)	Spot 3 (wt.%)	Spot 4 (at.%)	Spot 4 (wt.%)	Area 1 (at.%)	Area 1 (wt.%)
O	1	0.6	-	-	-	-	-	-	-	-
Mg	60.8	57.1	2.9	2.5	2.7	2.2	25.8	15.5	10.8	6
Al	0.1	0.1	93.6	89.4	81.2	73.4	36.6	24.4	45.1	28
Si	37.5	40.7	-	-	7.1	6.7	-	-	-	-
Mn	-	-	-	-	1.9	3.5	-	-	-	-
Fe	-	-	-	-	3.9	7.3	-	-	-	-
Cu	0.2	0.5	0.5	1.1	2.2	4.7	10.4	16.3	11.8	17,4
Zn	0.4	1	3	7	1	2.2	27.2	43.8	32.3	48.6

**Table 9:** Phases composition of the manufactured alloy in sample 1MH obtained by EDS.

Elements/Phases	Mg_2_Si (at.%)	Al-Base (at.%)	Eutectic (at.%)
Mg	61.7 ± 2.3	2.3 ± 1.4	11 ± 1
Al	0.1 ± 0	95.4 ± 2.1	44.2 ± 1.8
Si	37.6 ± 2.1	-	-
Cu	0.3 ±0.1	0.4 ± 0.2	10.7 ± 1.6
Zn	0.3 ± 0.1	1.9 ± 1.6	34.1 ± 1.9

**Table 10:** Microhardness and maximum compressive strength average values of the manufactured alloy in as-cast and heat-treated conditions.

Specimen	Hardness (HV0.2)	σmax (MPa)
1M	249 ± 14	588 ± 3
1MH	200 ± 11	495 ± 8
2M	258 ± 11	-
2MH	208 ± 15	-
1MH’	171 ± 7	426 ± 9

**Table 11:** Comparative table of microhardness values of the manufactured alloy in as-cast and heat-treated conditions and common cast aluminum alloys.

Alloy	Density (g/cm^3^)	Hardness Vickers	Ratio (Hardness/Density)
UNS A03080	2.79	80	28.7
UNS A07070	2.77	107	38.6
Current study (as-cast)	2.63	249	94.7
Current study (heat-treated 400 °C)	2.63	200	76
Current study (heat-treated 200 °C)	2.63	171	65

## Data Availability

Not applicable.
